# An Integrin-Dependent Role of Pouch Endoderm in Hyoid Cartilage Development

**DOI:** 10.1371/journal.pbio.0020244

**Published:** 2004-07-20

**Authors:** Justin Gage Crump, Mary E Swartz, Charles B Kimmel

**Affiliations:** **1**Institute of Neuroscience, University of OregonEugene, OregonUnited States of America

## Abstract

Pharyngeal endoderm is essential for and can reprogram development of the head skeleton. Here we investigate the roles of specific endodermal structures in regulating craniofacial development. We have isolated an *integrinα5* mutant in zebrafish that has region-specific losses of facial cartilages derived from hyoid neural crest cells. In addition, the cranial muscles that normally attach to the affected cartilage region and their associated nerve are secondarily reduced in *integrinα5^−^* animals. Earlier in development, *integrinα5* mutants also have specific defects in the formation of the first pouch, an outpocketing of the pharyngeal endoderm. By fate mapping, we show that the cartilage regions that are lost in *integrinα5* mutants develop from neural crest cells directly adjacent to the first pouch in wild-type animals. Furthermore, we demonstrate that Integrinα5 functions in the endoderm to control pouch formation and cartilage development. Time-lapse recordings suggest that the first pouch promotes region-specific cartilage development by regulating the local compaction and survival of skeletogenic neural crest cells. Thus, our results reveal a hierarchy of tissue interactions, at the top of which is the first endodermal pouch, which locally coordinates the development of multiple tissues in a specific region of the vertebrate face. Lastly, we discuss the implications of a mosaic assembly of the facial skeleton for the evolution of ray-finned fish.

## Abstract 2

A developmental mutant of the zebrafish is helping to uncover the processes by which the facial skeleton is patterned in vertebrates

## Introduction

The skeletal elements that form and support the vertebrate jaw and gills are derived from a specialized population of ectomesenchyme cells, the cranial neural crest (Platt 1893; Le Douarin 1982; Schilling and Kimmel 1994; but see Weston et al. 2004). In the larval zebrafish, crest cells of the first, or mandibular, arch give rise to Meckel's and palatoquadrate cartilages that constitute the lower and upper jaws, respectively. Several cartilages are derived from the second, or hyoid, arch, including the ceratohyal (CH) and hyosymplectic (HS) cartilages that support the jaw. In particular, the HS cartilage serves to connect the upper jaw to the skull by means of a hyomandibula (HM) plate and a symplectic (SY) anterior rod-like extension. In addition, the HM plate supports the overlying opercular apparatus that helps to ventilate the gills (Hughes and Shelton 1958). The tetrapod stapes is homologous to HM.

During vertebrate development, cranial neural crest cells delaminate from near the dorsal neural primordium and migrate to ventrolateral positions where they populate a series of pharyngeal arches (reviewed in Le Douarin 1982). Once the pharyngeal arches are established, skeletal elements develop from cylinders of neural crest whose mesodermal cores undergo stereotypic divisions to form the cranial muscles (Edgeworth 1935; Schilling and Kimmel 1997; Kimmel et al. 2001). The segmentally organized pharyngeal arches are separated from one another by reiterative outpocketings of the pharyngeal endoderm called pouches. Recent work in chicken has demonstrated an important role for endoderm in patterning cartilages of all the pharyngeal arches (Couly et al. 2002; Ruhin et al. 2003). Using grafting and ablation experiments, these researchers divided the pharyngeal endoderm into anterior-posterior (A-P) and mediolateral domains that are required for and have the ability to induce segment-specific pharyngeal cartilages. In zebrafish, experiments have demonstrated a genetic requirement for endoderm in pharyngeal cartilage development. *casanova* mutant embryos make no endoderm and pharyngeal cartilages fail to form, and transplantation experiments show that wild-type endoderm is sufficient to rescue cartilage development (Alexander et al. 1999; David et al. 2002). In addition, a role for pharyngeal pouches in segmentation and survival of cranial neural crest has been shown. In zebrafish *tbx1 (vgo)* mutant embryos, most pouches fail to develop and posterior pharyngeal cartilages are reduced and fused together (Piotrowski and Nusslein-Volhard 2000; Piotrowski et al. 2003). However, though endoderm is clearly critical for pharyngeal cartilage development, it is not well understood how interactions between neural crest–derived cells and endoderm produce segment-specific patterns of cartilage.

In this work, we isolate and characterize a zebrafish *integrinα5* loss-of-function mutant. Integrins are a family of heterodimeric receptors, composed of α and β subunits, that bind to ligands in the extracellular matrix such as fibronectin and laminin. Integrins have structural roles in adhesion that promote tissue integrity and cell migration, and signaling functions important for cell differentiation and survival (reviewed in Bokel and Brown 2002). Various studies in mouse and chick have shown a role for integrins and their ligands in neural crest migration. Integrins α4, α1, and αV are expressed early in neural crest development, and function-blocking antibodies against these integrins perturb crest migration in vitro (Delannet et al. 1994; Desban and Duband 1997; Kil et al. 1998; Testaz and Duband 2001). The in vivo roles of specific integrins in neural crest development are less clear (Yang et al. 1995; Gardner et al. 1996; Bader et al. 1998). Mice lacking Integrinα5, which in complex with primarily β1 forms the major fibronectin receptor, die early during embryogenesis because of mesodermal defects (Yang et al. 1993, 1999). Analysis of *integrinα5^−/−^* mouse embryos showed that Integrinα5 is required for the survival of a subset of hyoid crest (Goh et al. 1997). However, it was not known where Integrinα5 functions to control hyoid crest development.

Here we report that, in zebrafish, Integrinα5 functions in the pharyngeal endoderm to control hyoid crest development. In *integrinα5^−^* embryos, the first pharyngeal pouch fails to develop, and the lack of a first pouch correlates with reductions in specific regions of the HS cartilage. *integrinα5* mutants also have defects in a subset of dorsal first and second arch muscles and facial motor nerve VII, suggesting that Integrinα5 is required for region-specific development of multiple pharyngeal tissues. However, both expression and penetrance data suggest that the muscle and nerve defects are likely secondary to the cartilage and pouch defects. *integrinα5* is expressed in pharyngeal endoderm during pouch formation and is required in endoderm for both first pouch and hyoid cartilage development. In order to understand the remarkable specificity of the *integrinα5^−^* cartilage phenotype, we fate mapped regions of the HS cartilage in the hyoid arch. We found that the regions of the HS cartilage that are lost in *integrinα5* mutants develop from anterior crest–derived cells immediately adjacent to the first pouch. Analysis of *integrinα5* mutants suggests that the first endodermal pouch specifies a portion of the hyoid cartilage pattern by locally stabilizing hyoid crest. Lastly, we present a model in which new local interactions of endodermal structures with hyoid crest underlie the elaboration of the jaw support apparatus during the evolution of ray-finned fish.

## Results

### Isolation of a Mutation in Zebrafish *integrinα5*

**Figure 1:**
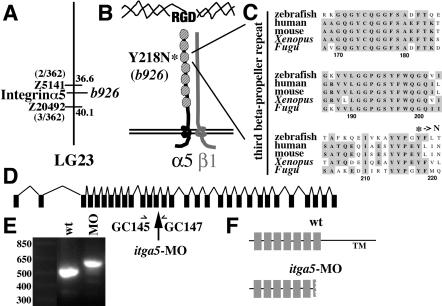
Identification of a Zebrafish *integrinα5* Mutant (A) Based on the hyoid cartilage phenotype the *b926* allele was mapped to LG23. Using polymorphism mapping, we placed *b926* between the markers Z5141 (2/362 recombinants/meioses) and Z20494 (3/362 recombinants/meioses). Databases of the partial zebrafish genomic sequence revealed that a homolog of *integrinα5* mapped to this region. (B) Zebrafish Integrinα5 protein is predicted to have seven extracellular beta-propeller repeats (stippled), a transmembrane domain, and a short intracellular cytoplasmic tail. Integrinα5 forms heterodimeric complexes with Integrin β chains, primarily β1, and binds extracellular matrix ligands containing the RGD motif. Sequencing of *integrinα*5 in *b926* revealed a T to A nucleotide substitution at position 952 of the cDNA that converts a tyrosine to an asparagine at amino acid 218, a residue within the third beta-propeller repeat. (C) Alignment of the third beta-propeller repeat of the Integrinα5 proteins of zebrafish, human, mouse, *Xenopus,* and *Fugu*. Y218 is absolutely conserved among all five species and is mutated to N in *b926*. (D) The genomic locus of *integrinα5* consists of 30 exons (not drawn to scale). The *integrinα*5 morpholino, *itga5*-MO, was designed against the exon13-intron splice site (arrow). (E) The primers GC145 and GC147 were used to amplify a 497-bp fragment from wild-type cDNA. PCR amplification was then performed using cDNA from 24-hpf embryos that had been injected with 10 ng of *itga5*-MO at the one-cell stage, the same concentration of morpholino used for our phenotypic analysis. The resultant product was 587 bp long, and sequencing confirmed that the increased length was due to the failure to splice out the intron between exons 13 and 14. The left lane of the agarose gel contains standard size markers in basepairs. (F) A schematic of the wild-type Integrinα5 protein shows seven extracellular repeats and the transmembrane (TM) domain. Inhibition of splicing by *itga5*-MO would be predicted to result in a nonfunctional protein that is truncated in the seventh repeat of the extracellular domain.

In a genetic screen for zebrafish with altered pharyngeal cartilages, we isolated a single recessive mutant allele, *b926,* with specific defects in the hyoid cartilage pattern. Using polymorphism mapping, we placed *b926* on Linkage Group (LG) 23 between the markers Z5141 and Z20492, a region containing a zebrafish *integrinα5* homolog (Figure 1A). We performed reverse transcription polymerase chain reaction (RT-PCR) to obtain a full-length cDNA encoding a protein product with 54% identity to mouse Integrinα5. Sequencing of *integrinα5* in *b926* revealed a T to A nucleotide substitution that segregated with the mutant phenotype. The *b926* mutation converts a conserved tyrosine residue to an asparagine in the third beta-propeller repeat of the extracellular domain, a region known to be important for ligand binding (Figure 1B and 1C) (Springer 1997; Mould et al. 2000). In addition, a morpholino designed against the exon13-intron splice site of *integrinα5* (*integrinα5*-MO; Figure 1D) phenocopies both the cartilage and first pouch defects of *b926*. We confirmed by RT-PCR that *itga5*-MO effectively inhibits the splicing of *integrinα5* (Figure 1E and 1F). We conclude that *b926* is a loss-of-function mutation in zebrafish *integrinα5*.

### Region-Specific Pharyngeal Defects in *integrinα5* Mutants

**Figure 2:**
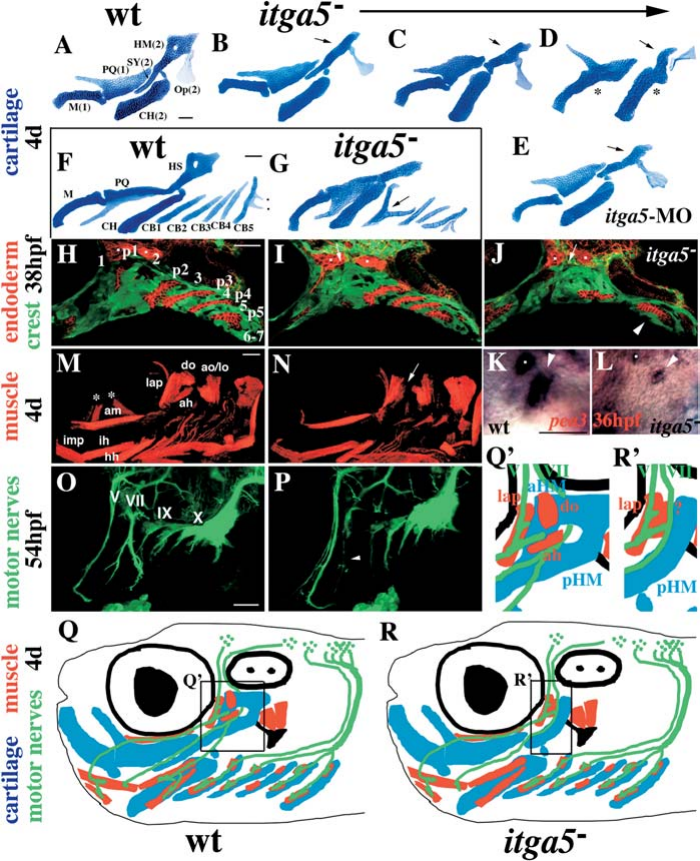
Region-Specific Pharyngeal Defects in *integrinα5* Mutants (A–E) Flat mount dissections of hyoid and mandibular cartilages from fixed, 4-d-old wild-type (A), *integrinα5^−^* (B–D), and *itga5*-MO (E) animals. Meckel's (M) and palatoquadrate (PQ) cartilages are derived from the mandibular arch (1), and CH, SY, and HM cartilages and the opercle (Op) bone are derived from the hyoid arch (2). A phenotypic series (B–D) shows that the anterior half of HM (arrows) is absent and SY is progressively reduced in *integrinα5^−^* animals. Rarely, mandibular and hyoid joints are also missing in *integrinα5^−^* animals (asterisks in D). (E) Animals treated with *itga5*-MO display similar reductions of HM (arrow) and SY. (F and G) Flat-mount dissections of the pharyngeal cartilages of 4-d-old wild-type (F) and *integrinα5^−^* (G) animals. In addition to the mandibular and hyoid cartilages, the five CB cartilages (CB1–CB5) that are derived from the third through seventh arches are shown. Note the teeth on CB5 (dots in F). In *integrinα5^−^* embryos we see rare fusions of CB cartilages (arrow in G). (H–J) Confocal micrographs of the pharyngeal arches of wild-type *fli1*-GFP (H) and *integrinα5^−^; fli1*-GFP (I and J) embryos stained with anti-GFP and Zn8 antibodies at 38 hpf. Neural crest cells of the pharyngeal arches are labeled with *fli1*-GFP (green, numbered in [H]), and the pharyngeal pouches are labeled by the Zn8 antibody (red, numbered p1–p5 in [H]). In *integrinα5^−^; fli1*-GFP embryos, the first pouch is absent or very reduced at 38 hpf (arrows in I and J). Less frequently, we also see reductions in more posterior pouches in *integrinα5^−^; fli1*-GFP embryos (arrowhead in J shows a single endodermal mass where p3–p5 would be in wild-type embryos). The Zn8 antibody also recognizes cranial sensory ganglia (dots). (K and L) In situ hybridizations of wild-type (K) and *integrinα5^−^* (L) embryos stained with the pharyngeal pouch marker *pea3* at 36 hpf (arrowhead denotes first pouch). The first pouch of *integrinα5^−^* embryos is very reduced, but still expresses *pea3*. Sensory ganglia also stain with *pea3* (dots). (M and N) Cranial muscles of 4-d-old wild-type *fli1*-GFP (M) and *integrinα5^−^; fli1*-GFP (N) embryos stained with MF20 antibody. Mandibular muscles (intermandibularis posterior [imp], adductor mandibulae [am], levator arcus palatine [lap], and do) and hyoid muscles (interhyal , hyohyal [hh], ah, ao, and lo) are labeled in wild-type. *integrinα5^−^* embryos have a selective reduction of do and ah muscles (arrow in [N]). Confocal projections of *integrinα5^−^* animals did not include ocular muscles (asterisks in M). (O and P) Cranial motor nerves of wild-type *islet1*-GFP (O) and *integrinα5^−^*; *islet1*-GFP (P) live embryos at 54 hpf. *islet1*-GFP-expressing cranial motor neurons innervate muscles of the pharyngeal arches with the following strict segmental correspondence: trigeminal (V)—mandibular; facial (VII)—hyoid; glossopharyngeal (IX)—third; and vagus (X)—fourth through seventh. In *integrinα5^−^*; *islet1*-GFP embryos, facial nerve VII (arrowhead in P) is reduced and/or fails to branch. (Q and R) Summary of *integrinα5* regional pharyngeal defects extrapolated to a 4-d-old embryo and color-coded for cartilage (blue), muscle (red), and nerve (green). Shown in black are the eye (filled circle within larger circle), ear (two dots within oval), and opercle bone (mushroom). In wild-type animals, facial nerve VII innervates and passes by do and ah muscles that are in close association with the aHM cartilage region (enlarged in Q′). In *integrinα5* mutants, we see specific reductions of the first pouch (not shown), the aHM cartilage region, do and ah muscles, and facial nerve VII (enlarged in R′). Scale bars: 50 μm.

*integrinα5* mutant zebrafish showed partially penetrant and variably expressive losses of specific hyoid cartilage regions at 4 d (Figure 2; Table 1). The most frequent phenotype seen in *integrinα5^−^* animals was a specific loss of the anterior half of the HM plate (anterior HM [aHM]) (Figure 2B). In other *integrinα5* mutants, we saw variable reductions of the SY element in addition to aHM (Figure 2C). In what we interpret as the most severe *integrinα5 (b926)* phenotype, HS was reduced to a rod that variably fused with CH; rarely, the first arch joint was fused as well (Figure 2D). However, even in the most severe class of *integrinα5* mutants, the posterior half of HM (posterior HM [pHM]), the CH, and the hyoid opercle bone remained unaffected. In addition, *integrinα5* mutants had variably reduced numbers of ceratobranchial (CB) cartilages and rare fusions of adjacent CBs (Figure 2G; Table 1). Animals injected with *itga5*-MO displayed a similar range and spectrum of cartilage phenotypes (Figure 2E; Table 1).

**Table 1:**
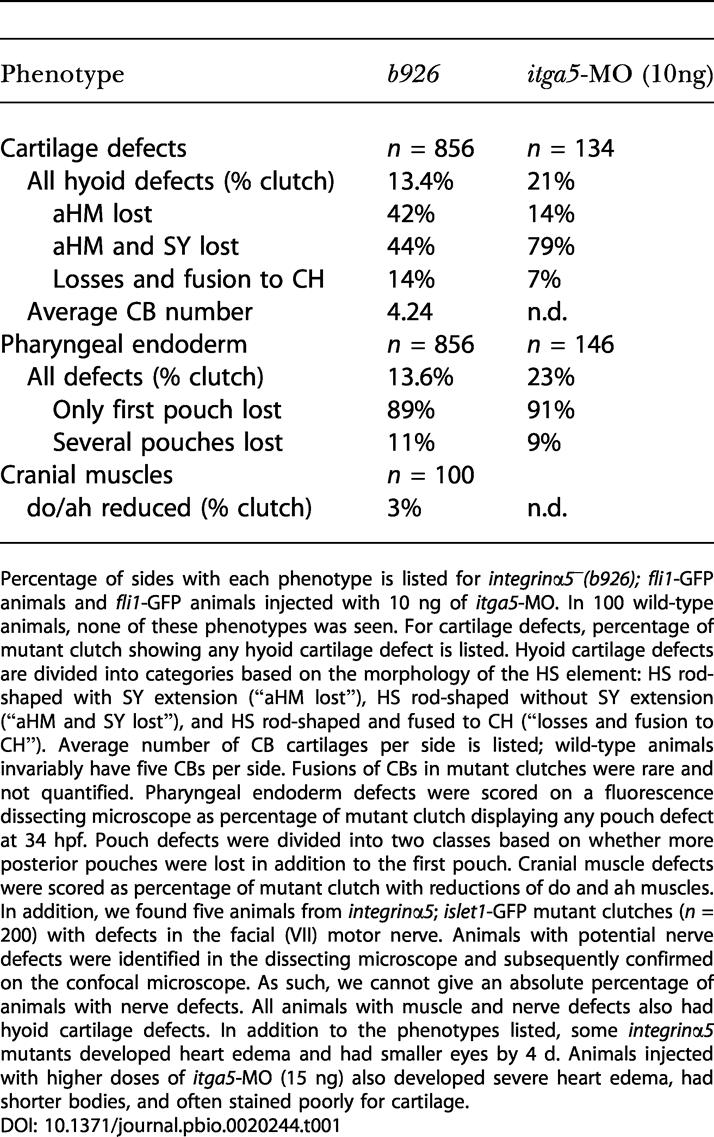
Pharyngeal Defects in Animals Reduced for *integrinα5*

Percentage of sides with each phenotype is listed for *integrinα5^−^(b926); fli1*-GFP animals and *fli1*-GFP animals injected with 10 ng of *itga5*-MO. In 100 wild-type animals, none of these phenotypes was seen. For cartilage defects, percentage of mutant clutch showing any hyoid cartilage defect is listed. Hyoid cartilage defects are divided into categories based on the morphology of the HS element: HS rod-shaped with SY extension (“aHM lost”), HS rod-shaped without SY extension (“aHM and SY lost”), and HS rod-shaped and fused to CH (“losses and fusion to CH”). Average number of CB cartilages per side is listed; wild-type animals invariably have five CBs per side. Fusions of CBs in mutant clutches were rare and not quantified. Pharyngeal endoderm defects were scored on a fluorescence dissecting microscope as percentage of mutant clutch displaying any pouch defect at 34 hpf. Pouch defects were divided into two classes based on whether more posterior pouches were lost in addition to the first pouch. Cranial muscle defects were scored as percentage of mutant clutch with reductions of do and ah muscles. In addition, we found five animals from *integrinα5*; *islet1*-GFP mutant clutches (*n =* 200) with defects in the facial (VII) motor nerve. Animals with potential nerve defects were identified in the dissecting microscope and subsequently confirmed on the confocal microscope. As such, we cannot give an absolute percentage of animals with nerve defects. All animals with muscle and nerve defects also had hyoid cartilage defects. In addition to the phenotypes listed, some *integrinα5* mutants developed heart edema and had smaller eyes by 4 d. Animals injected with higher doses of *itga5*-MO (15 ng) also developed severe heart edema, had shorter bodies, and often stained poorly for cartilage

In addition to cartilage defects, we found partially penetrant reductions of the first pharyngeal pouch, an endodermal outpocketing, in *integrinα5* mutants (Figure 2I and 2J). Loss of the first pouch was apparent as early as 24 hours post fertilization (hpf), and similar first pouch reductions were seen in animals injected with *itga5*-MO (data not shown; Table 1). Although the majority of *integrinα5^−^* embryos showed defects restricted to the first pouch, a few *integrinα5^−^* embryos had graded reductions of more posterior pouches as well (Figure 2J; Table 1). In order to examine whether the reduced first pouches in *integrinα5^−^* embryos retained pouch identity, we examined expression of the pharyngeal pouch marker *pea3,* a downstream effector of Fgf signaling (Figure 2K) (Roehl and Nusslein-Volhard 2001). We found that the reduced first pouches of *integrinα5* mutants still expressed *pea3* at 36 hpf (Figure 2L).

As both the pouch and cartilage phenotypes were incompletely penetrant in *integrinα5* mutants, often with one side of an embryo showing defects and the other side not, we examined how tightly coupled the phenotypes were in individual sides. In order to visualize early pharyngeal arch structure in live animals, we made use of a *fli1–*green fluorescent protein (GFP) transgenic line (Lawson and Weinstein 2002). *fli1*-GFP expression initiates in neural crest cells of the pharyngeal arches shortly after crest migration (ca. 18 hpf) and persists as pharyngeal cartilages and bones develop. Pouches are evident as nonexpressing regions between the GFP-labeled crest-derived cells of the arches. We sorted live *integrinα5^−^* animals for first pouch defects at 36 hpf and then grew these animals to 4 d to analyze pharyngeal cartilages. Strikingly, we found a strong correlation between reductions of the first pouch and later hyoid cartilage defects in *integrinα5* mutants. *integrinα5^−^* sides that lacked the first pouch early had hyoid cartilage defects 93% of the time (*n =* 116), whereas only 7% of *integrinα5^−^* sides with a normal first pouch early developed hyoid cartilage defects (*n =* 108). The correlation was highly significant (*p* < 0.0001).

We next asked whether Integrinα5 was required for the development of cranial muscles. By 4 d of development, the mesoderm of the first pharyngeal arch has undergone stereotypic subdivisions to form, ventrally, intermandibularis anterior and intermandibularis posterior; medially, adductor mandibulae; and, dorsally, levator arcus palatini and dilatator operculi (do) cranial muscles. Second arch mesoderm gives rise to interhyal and hyohyal muscles, ventrally, and adductor hyomandibulae (ah), adductor operculi (ao), and levator operculi (lo) muscles, dorsally (Edgeworth 1935; Schilling et al. 1997; Figure 2M). In a few *integrinα5* mutants, the dorsal first and second arch muscles, do and ah, were selectively reduced, whereas levator arcus palatini, ao, and lo were present but appeared closer together (Figure 2N; Table 1). The muscles that were disrupted in *integrinα5* mutants correspond to those that associate most closely with the aHM cartilage (schematized in Figure 2Q and 2R).

Lastly, we found that *integrinα5* mutants had specific defects in the nerve that innervates second arch muscles. Facial motor neurons send a ventral-directed nerve (VII) that innervates dorsal second arch muscles ah and ao, passes through the foramen of HM, and subsequently branches to innervate ventral muscles interhyal and hyohyal (Higashijima et al. 2000; Figure 2O). In a small fraction of *integrinα5* mutants, facial nerve VII failed to branch and/or was hypotrophic (Figure 2P). Both nerve and muscle defects were only seen in *integrinα5* mutants that also displayed cartilage defects. Moreover, the lower penetrance of nerve and muscle defects suggests that they are secondary to endodermal and/or crest defects. In conclusion, we have found a requirement for Integrinα5 in the development of multiple pharyngeal tissues in the vicinity of the first pouch and aHM cartilage region.

### *integrinα5* Is Expressed Dynamically in Pharyngeal Endoderm and Cranial Neural Crest

**Figure 3:**
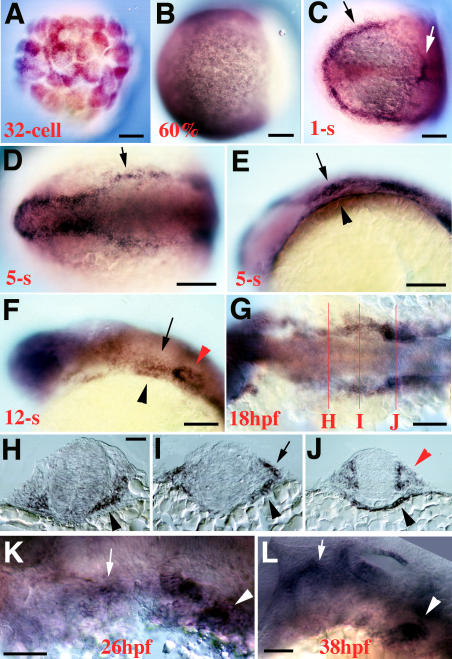
*integrinα5* Expression in Pharyngeal Endoderm and Cranial Neural Crest (A) At the 32-cell stage, strong maternal *integrinα5* expression is seen. (B) At 60% epiboly, *integrinα5* expresses broadly throughout the mesendoderm. (C) Dorsal view of a 1-s-stage embryo. *integrinα5* transcript is concentrated in the ectoderm at the edge of the neural plate (black arrow), in scattered presumptive endodermal cells, and in the first somite (white arrow). (D and E) Dorsal (D) and lateral (E) views of a 5-s-stage embryo show ectodermal (arrows) and pharyngeal endodermal (arrowhead) expression domains of *integrinα5*. Ectodermal *integrinα5* expression includes migratory hyoid crest, otic placode, and forebrain. (F) At the 12-s stage, *integrinα5* continues to be expressed in the pharyngeal endoderm (black arrowhead), postmigratory hyoid crest (arrow), ear (red arrowhead), and forebrain. (G–J) At 18 hpf, a dorsal view of an embryo stained for *integrinα5* transcript (G) shows approximate axial levels at which cross-sections were prepared. (H) A cross-section at the level of the first pouch shows strong *integrinα5* expression in the pharyngeal endoderm (arrowhead). (I) A cross-section at the level of the hyoid arch shows expression of *integrinα5* in neural crest (arrow) and pharyngeal endoderm (arrowhead). (J) A cross-section at the level of the ear shows *integrinα5* expression in the otic epithelium (red arrowhead) and pharyngeal endoderm (black arrowhead). (K and L) At 26-hpf (K) and 38-hpf (L) stages, *integrinα5* transcript is enriched in the region of the most recent forming pharyngeal pouch (arrowheads) and in patches of crest (arrows). Scale bars: (A–C), (F), and (G): 100 μm; (D), (E), and (H–L): 50 μm.

In order to understand where Integrinα5 might be acting to control pharyngeal arch development, we examined the expression of *integrinα5* mRNA by in situ hybridization. The *integrinα5* expression domains were complex and dynamic, and we do not give an exhaustive description of domains other than the pharynx here. We observed *integrinα5* expression as early as the 32-cell stage, indicating a maternal *integrinα5* contribution (Figure 3A). Maternal expression of *integrinα5* also has been reported in frog (Whittaker and DeSimone 1993). By gastrulation stage (60% epiboly), the mesendoderm broadly expressed *integrinα5* (Figure 3B). At the 1-somite (s) stage, *integrinα5* expression was in ectoderm adjacent to the anterior neural plate, a domain consistent with cranial neural crest and placode precursors (Figure 3C). In addition, we saw strong expression in the first somite and posterior mesoderm and weaker expression in scattered, large cells lateral and anterior to the notochord that we interpret as early endoderm (Warga and Nusslein-Volhard 1999). By 5-s stages, cranial neural crest and the otic placode expressed *integrinα5* (Figure 3D), and pharyngeal endoderm expression was seen ventrally along the surface of the yolk (Figure 3E). From 12-s to 18-s (18 hpf), pharyngeal endoderm continued to express *integrinα5,* and the ectodermal expression domain became more restricted to hyoid crest–derived tissue and the otic placode (Figure 3F and 3G). Endoderm and ectoderm expression domains of *integrinα5* were apparent most clearly in 18 hpf cross-sections (Figure 3H–3J). In particular, we observed strong *integrinα5* expression throughout pharyngeal endoderm, including the first pouch (Figure 3H). At later times, we saw dynamic *integrinα5* expression in both crest derivatives and pharyngeal endoderm. The six pharyngeal pouches form in an anterior to posterior wave of development. By 26 hpf, the fourth pouch, which was the most posterior pouch forming at this time (J.G.C. and C.B.K., unpublished data), expressed *integrinα5,* whereas *integrinα5* expression was no longer seen in the first pouch (Figure 3K). At 38 hpf, the sixth pouch, the last to form, strongly expressed *integrinα5,* yet the fourth pouch was nonexpressing (Figure 3L). In addition, dynamic *integrinα5* expression was seen in patchy zones of pharyngeal crest. Finally, *integrinα5* expression was not affected by the *b926* mutation (examined at 12-s; data not shown). In conclusion, *integrinα5* expression in the pharyngeal endoderm is in a pattern that both spatially and temporally corresponds to regions of pouch formation, and expression of *integrinα5* in the crest begins during premigratory stages and later becomes refined to patches of crest derivatives within the pharyngeal arches.

### Integrinα5 Is Required in Endoderm but Not Crest for First Pouch and Hyoid Cartilage Development

**Figure 4:**
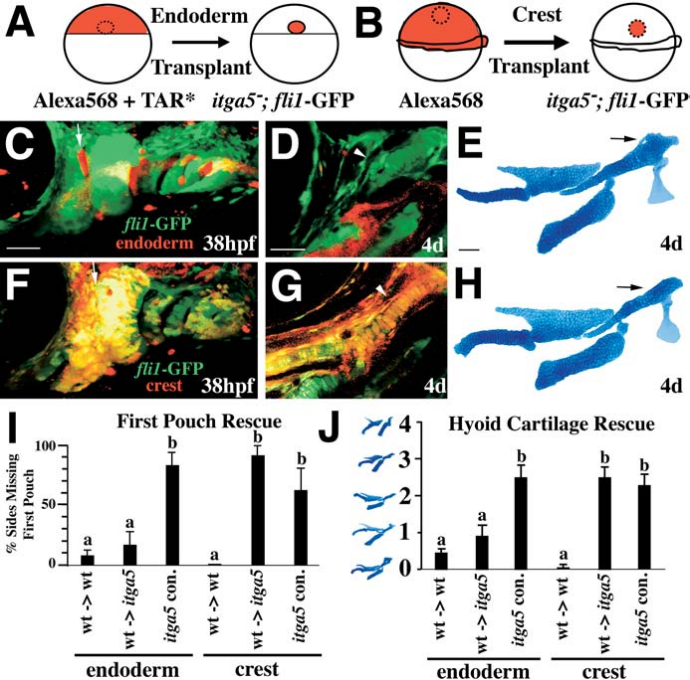
*integrinα5* Requirement in Endoderm but Not Crest (A and B) Schematics of endoderm (A) and crest (B) transplant experiments. (C–E) In endoderm transplants, confocal projections at 38 hpf (C) and 4 d (D) of a single *integrinα5^−^; fli1*-GFP host animal show that wild-type TAR* donor tissue contributed efficiently to pharyngeal endoderm (red) but not crest (green). (E) Flat-mount dissection of mandibular and hyoid cartilages from the individual in (C) and (D). Wild-type TAR* endoderm rescues first pouch development (arrow in [C]) and partially rescues hyoid cartilage development (arrowhead in [D] and arrow in [E]) in *integrinα5^−^* embryos. (F–H) In crest transplants, confocal projections of a single *integrinα5^−^; fli1*-GFP host animal show extensive colocalization (yellow) of donor tissue (red) with crest (green) at 38 hpf (F) and 4 d (G). Donor tissue does not contribute to endoderm or mesoderm. (H) Flat-mount dissection of mandibular and hyoid cartilages from the individual in (F) and (G). Neither the first pouch defects (arrow in [F]) nor hyoid cartilage defects (arrowhead in [G], arrow in [H]) of *integrinα5^−^* animals were rescued by wild-type crest. (I and J) Wild-type and *integrinα5^−^* sides that received wild-type endoderm (*n_wt_* = 39; *n_itga5_* = 12) or crest (*n_wt_* = 30; *n_itga5_* = 12) transplants are plotted against the contralateral *integrinα5^−^* control sides that did not receive transplants. (I) First pouch defects are quantified as percent of sides missing the first pouch. For endoderm transplants, *integrinα5^−^* recipient sides were rescued to wild-type levels. For crest transplants, *integrinα5^−^* recipient sides were not rescued compared to control sides. (J) Hyoid cartilage defects are quantified according to a mutant cartilage index: 0, wild-type; 1, partial aHM reduction; 2, full aHM loss; 3, aHM and SY losses; and 4, aHM and SY losses and fusion to CH. For endoderm transplants, *integrinα5^−^* recipient sides were rescued to wild-type index. For crest transplants, *integrinα5^−^* recipient sides were not rescued compared to control sides. Lowercase letters (a, b) in plots designate statistically significant groupings using Tukey-Kramer HSD test. Scale bars: 50 μm.

As *integrinα5* expression was observed in both pharyngeal endoderm and neural crest, we used transplantation experiments to determine in which tissues Integrinα5 was required for first pouch and hyoid cartilage development (Figure 4). Since it is difficult to transplant large amounts of endoderm from normal zebrafish embryos, we used forced expression of the activated Taram-A receptor (TAR*) to generate donor embryos consisting almost entirely of endoderm (see David et al. [2002] for details). This method allows the specific and unilateral introduction of large amounts of endoderm into mutant hosts. Transplants were performed at 40% epiboly (late blastula; ca. 4 hpf), and the first pouch was scored at 38 hpf in live animals (Figure 4A). In wild-type to wild-type control transplants, 8% of recipient sides developed first pouch defects, suggesting a low level of toxicity of the TAR* construct. We then transplanted wild-type TAR* endoderm into *integrinα5^−^; fli1*-GFP hosts. Control mutant sides that did not receive donor endoderm had first pouch defects in 83% of cases. In contrast, first pouch defects were seen in only 17% of mutant sides that received wild-type donor endoderm (Figure 4C and 4D; summarized in Figure 4I). Thus, wild-type endoderm was able to rescue pouch formation in *integrinα5* mutants. Furthermore, this rescue was dependent on donor endoderm contributing to the first pouch.

We next asked whether wild-type endoderm also was able to rescue hyoid cartilage defects in *integrinα5^−^* embryos. In order to quantify the severity of hyoid defects in *integrinα5* mutants, we devised a mutant cartilage index that ranged from zero for wild-type to four for the most severe hyoid defects (see legend for Figure 4). In wild-type to wild-type control transplants, the mutant cartilage index was 0.46, consistent with TAR* causing a low level of defects on its own. The control nonrecipient sides of *integrinα5^−^; fli1*-GFP animals had an index of 2.5. In contrast, the index of mutant sides that received the TAR* endoderm transplant was rescued to 0.92 (Figure 4D and 4E; summarized in Figure 4J). Hence, wild-type endoderm can nonautonomously rescue hyoid cartilage development in *integrinα5* mutants.

We also tested whether Integrinα5 was required only in crest for first pouch and hyoid cartilage development. We modified the hindbrain transplantation technique described in Maves et al. (2002) to transplant neural crest precursor cells at shield stages (Figure 4B). In wild-type to wild-type controls, transplants resulted in donor cells constituting a large proportion of the crest cells within the pharyngeal arches and resultant cartilages. In contrast to wild-type endoderm rescue, introduction of substantial amounts of wild-type crest failed to rescue first pouch formation in *integrinα5^−^; fli1*-GFP animals (Figure 4F and 4G; summarized in Figure 4I). Furthermore, transplanted wild-type crest did not improve the mutant cartilage index of *integrinα5^−^; fli1*-GFP animals (2.50 for recipient sides and 2.29 for nonrecipient sides) (Figure 4G and 4H; summarized in Figure 4J). Thus, wild-type crest was not able to rescue first pouch and hyoid cartilage development in *integrinα5* mutants.

### Fate Map of Hyoid Cartilages

**Figure 5:**
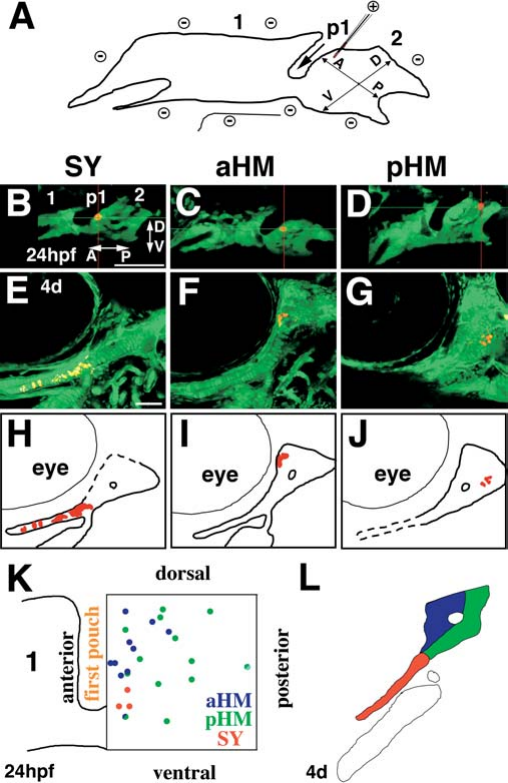
Fate Map of Hyoid Cartilages (A) In in vivo microelectroporation, a glass needle coupled to a positive electrode and filled with Alexa568 amine dextrans (red) is positioned in the hyoid arch (2) of wild-type *fli1*-GFP embryos immobilized adjacent to a negative electrode. A short pulse of current delivers dye into single or pairs of cells. A-P and D-V axes, the mandibular arch (1), and first pouch (p1) are designated in (A) and (B). (B–D) Confocal sections of *fli1*-GFP-labeled hyoid arches (2) (green) show the positions of Alexa568-labeled cells (red) shortly after microelectroporation (24 hpf). (E-J) At 4 d, confocal micrographs (E–G) (schematized in [H–J]) show the resultant fate of labeled crest cells (red) in the hyoid cartilage regions (green). Examples shown include labeled hyoid cells that contributed exclusively to SY (B, E, and H), aHM (C, F, and I), and pHM (D, G, and J) cartilage regions. (K) The relative distances (normalized to one) of hyoid crest cells at 24 hpf that contributed to SY (red), aHM (blue), and pHM (green) regions are plotted along A-P and D-V axes. The first pouch and partial outline of the mandibular arch (1) are drawn for reference. One cell gave rise to an aHM/pHM (blue/green) mixed lineage, and another cell gave rise to pHM and unidentified cells (green/light blue). SY and aHM progenitors map to more anterior domains (i.e., closer to the first pouch) than do pHM progenitors (relative distances from anterior: SY, 0.12 ± 0.11; aHM, 0.17 ± 0.06; pHM 0.43 ± 0.05; statistically different using Tukey-Kramer HSD test). SY progenitors map to a more ventral domain than do aHM and pHM progenitors (relative distances from dorsal: SY, 0.68 ± 0.11; aHM, 0.33 ± 0.06; pHM 0.37 ± 0.05; statistically different using Tukey-Kramer HSD test). No significant differences along the mediolateral axis were seen between regions (relative distances from lateral: SY, 0.47 ± 0.12; aHM, 0.53 ± 0.07; pHM 0.43 ± 0.06). (L) For the fate analysis, the 4-d HS cartilage was subdivided into SY (red), aHM (blue), and pHM (green) regions. The outline of the CH cartilage is also shown. Scale bars: 50 μm.

Understanding the developmental basis for the specificity of the *integrinα5^−^* cartilage phenotype requires a fate map of pharyngeal cartilages in wild-type animals. Here we focus on the origins of the SY, aHM, and pHM regions of HS, and a more complete mandibular and hyoid fate map will be published elsewhere. We used in vivo microelectroporation (Lyons et al. 2003) to label crest cells at 24 hpf and later monitor their cartilage fate (see Materials and Methods; Figure 5A). Representative examples of 24-hpf in vivo microelectroporations show cells that contributed to SY, aHM, or pHM regions at 4 d (Figure 5B–5J). We plotted the origins of cells that contribute to each region along normalized A-P and dorsal-ventral (D-V) axes (Figure 5K). A comparison of mean distances along the A-P axis showed that cells that contributed to aHM and SY clustered on average 6–7 μm, or 1–2 cell diameters, from the first pouch (the anterior border of the arch). In contrast, cells contributing to pHM were on average 16 μm, or three cell diameters, away from the first pouch. A comparison of mean distances along the D-V axis showed that cells contributing to SY were more ventral than cells contributing to aHM and pHM. No statistically significant differences along the mediolateral axis were seen between cells contributing to different HS regions (data not shown; see legend of Figure 5). We conclude that HS cartilage regions most sensitive to loss of Integrinα5 are those developing just beside the first pouch.

### Increased Cell Death and Disorganized *goosecoid* Expression in the Hyoid Arches of *integrinα5^−^* Embryos

**Figure 6:**
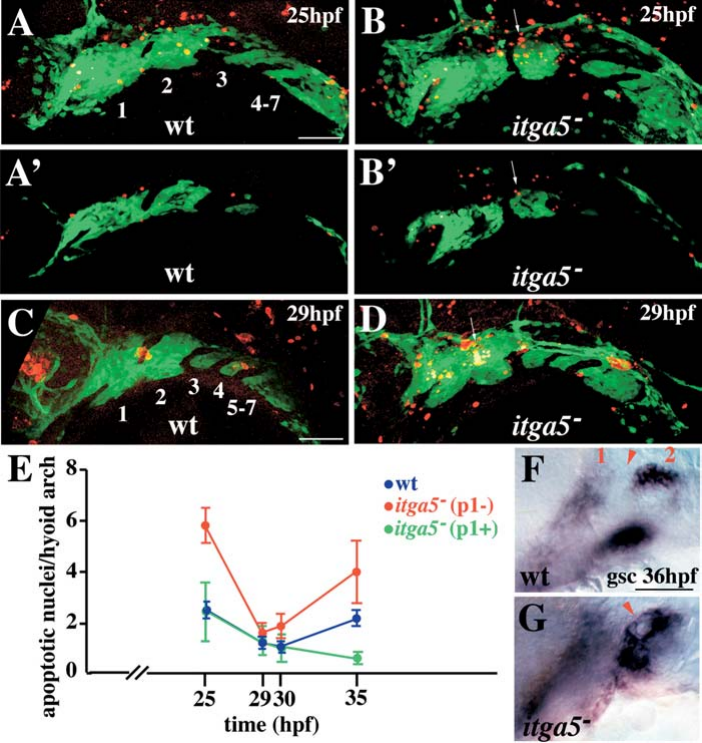
Increased Apoptosis and Disorganized *gsc* Expression in the Hyoid Arches of *integrinα5* Mutants (A–D) TUNEL staining of wild-type *fli1-*GFP (A and C) and *integrinα5^−^; fli1*-GFP (B and D) animals shows apoptotic nuclei (red) relative to the GFP-expressing crest of the pharyngeal arches (green) at 25 hpf (A and B) and 29 hpf (C and D). In wild-type confocal projections arches are numbered. (A′) and (B′) are representative confocal sections taken from the projections in A and B. In *integrinα5^−^* animals lacking the first pouch, increased apoptosis (arrows in [B] and [D]) is seen in the dorsal anterior hyoid arch adjacent to where the first pouch would be in wild-type animals. In mutant sections (B′), TUNEL-positive cells (arrow) colocalize with the *fli1*-GFP crest marker. (E) The number of apoptotic nuclei per hyoid arch is plotted versus time for wild-type sides (blue) and *integrinα5^−^* sides without (p1−; red) or with (p1+; green) a normal first pouch. At 25 hpf, *integrinα5^−^* hyoid arches had more apoptotic nuclei than wild-type hyoid arches only when the first pouch was defective (*p* < 0.0001). At later time points, *integrinα5^−^* hyoid arches missing the first pouch had a tendency to have more apoptotic nuclei than wild-type or *integrinα5^−^* arches with normal first pouches (only *itga5^−^* with a normal first pouch versus *itga5^−^* without at 35 hpf is statistically significant, *p* < 0.05). Total sides examined: 25 hpf: *n_wt_* = 40, *n_itga5_* = 38; 29 hpf: *n_wt_* = 30, *n_itga5_* = 26; 30 hpf: *n_wt_* = 30, *n_itga5_* = 20; and 35 hpf: *n_wt_* = 30, *n_itga5_* = 14. (F and G) *gsc* expression at 36 hpf labels dorsal and ventral domains of hyoid crest. Mandibular (1) and hyoid (2) arches are numbered, and the first pouch is denoted by arrowhead. In wild-type animals, dorsal and ventral hyoid *gsc* domains are well separated. In this *integrinα5^−^* animal, dorsal and ventral hyoid *gsc* domains are fused, and disorganized *gsc*-expressing cells envelop the reduced first pouch (arrowhead). Scale bars: 50 μm.

We next investigated whether the losses of aHM and SY regions in *integrinα5^−^* embryos correlated with increased cell death in the hyoid arch (Figure 6). At 25 hpf, TUNEL staining revealed a greater than 2-fold increase in apoptosis over wild-type in the hyoid arches of *integrinα5^−^; fli1*-GFP embryos (Figure 6A, 6B, and 6E). A moderate tendency toward increased apoptosis was also seen in the hyoid arches of *integrinα5^−^; fli1*-GFP embryos from 29 to 35 hpf (Figure 6D and 6E). Apoptotic nuclei appeared to cluster in the dorsal anterior portion of the hyoid arch (Figure 6B and 6D), and colocalization with *fli1-*GFP, a marker of neural crest, in confocal sections showed that some of these nuclei corresponded to dying crest cells (Figure 6B′). Interestingly, we observed an increase in hyoid apoptosis only in *integrinα5^−^; fli1*-GFP sides in which the first pouch failed to develop (Figure 6E). In addition, at 14 hpf and 18 hpf, stages before which the first pouch has normally fully formed, no increase in cell death was seen in the cranial neural crest of *integrinα5^−^* animals (data not shown). These results are consistent with the first pouch being required for the survival of hyoid crest that contributes to aHM and SY.

We also examined whether hyoid crest was correctly specified in *integrinα5* mutant embryos. Hyoid crest expresses Hox class 2 genes, whereas mandibular crest is Hox nonexpressing (Hunt et al. 1991). No defects were seen in the expression of *hoxa2* in the hyoid arches of *integrinα5* mutants at 36 hpf (data not shown). In 36-hpf wild-type animals, *goosecoid (gsc)* expression marks dorsal and ventral domains within the hyoid arch (Figure 6F). In *integrinα5^−^* embryos, *gsc* domains were present, although in 12% of mutants they were variably disorganized. In the example shown in Figure 6G, the dorsal hyoid *gsc* domain was disorganized and fused to the ventral hyoid *gsc* domain. However, as the *gsc* defects were of significantly lower penetrance than the cartilage defects, we conclude that the majority of specific cartilage defects seen in *integrinα5^−^* embryos are not due to altered *gsc* expression.

### A Subset of Hyoid Crest Shows Aberrant Behavior and Does Not Contribute to Cartilage in *integrinα5* Mutants

**Figure 7:**
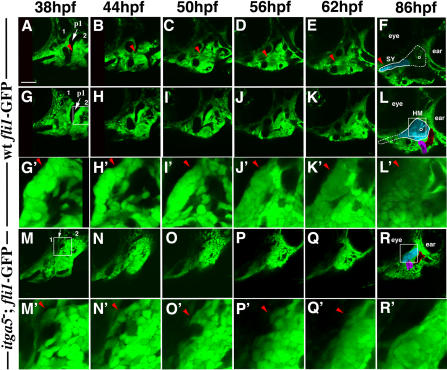
Anterior Hyoid Crest Cells Display Aberrant Behavior in *integrinα5* Mutants Confocal time-lapse recordings show hyoid cartilage development in wild-type *fli1*-GFP (Videos S1 and S2) and *integrinα5^−^; fli1*-GFP (Video S3) animals from 38 hpf to 86 hpf (*n_wt_* = 3; *n_itga5_* = 4). Videos S1 and S2 are different depths of the same time-lapse recording. Representative imaging stills of Video S1 (A–F), Video S2 (G–L), and Video S3 (M–R) were taken at 38 hpf (A, G, and M), 44 hpf (B, H, and N), 50 hpf (C, I , and O), 56 hpf (D, J, and P), 62 hpf (E, K, and Q), and 86 hpf (F, L, and R). At the beginning of the recordings (A, G, and M), the mandibular (1) and hyoid (2) arches are numbered and an arrow denotes the first pouch (p1). At the end of the recordings (F, L, and R), the cartilage regions are clearly visible as large cells with thick matrix (pseudocolored blue). The outline of the HS cartilage, a composite of SY and HM regions, is shown in (F) and (L). As a reference, the opercle bone and ao/lo hyoid muscle mass are pseudocolored purple and red, respectively, and the eye and ear are labeled. In Video S1 (A–F), red arrowheads denote a cluster of cells adjacent to the first pouch that undergo cellular rearrangements and form the long, anterior SY extension in wild-type animals. (G′–R′) show magnifications of HM-forming regions taken from (G–R) and correspond to areas within white boxes given in (G) and (L) for (G–L) and in (M) and (R) for (M–R). In wild-type development, hyoid crest cells adjacent to the first pouch remain a tightly packed mass as aHM chondrifies (e.g., cells denoted by red arrowheads in G′–L′). In *integrinα5* mutants, the first pouch is missing (white arrow in [M]), and anterior hyoid crest cells are disorganized at 38 hpf (e.g., arrowhead in [M′]). Over time, anterior hyoid crest cells migrate out of the region and do not contribute to cartilage (e.g., arrowheads in [N′–Q′]). In contrast, the pHM region and the opercle bone develop normally from more posterior hyoid crest in *integrinα5^−^* animals (R). Scale bar: 50 μm.

The first pharyngeal cartilages begin to chondrify around 48 hpf (Schilling and Kimmel 1997). In order to understand neural crest cell behavior during cartilage formation in wild-type animals, we used the *fli1*-GFP line to make extended time-lapse recordings of hyoid arch development that began at 38 hpf and ended at 86 hpf, an endpoint when cartilage elements are readily identifiable (Figure 7). In one focal plane, the SY region was observed to form from tightly packed cells adjacent to the ventral tip of the first pouch at 38 hpf (Video S1; Figure 7A–7F). In another focal plane, aHM was observed to form from a tightly packed mass of *fli1*-GFP-labeled cells located directly adjacent to the first pouch in the dorsal, anterior portion of the hyoid arch at 38 hpf (Video S2; Figure 7G–7L). We found that crest cells that contributed to aHM remained fairly static during the period of observation, though local rearrangements that contribute to the flattening of the HM plate were not analyzed in detail here (enlarged in Figure 7G′–7L′). The pHM region formed from cells located posterior to the aHM mass at 38 hpf. In general, our time-lapse recordings of wild-type hyoid development supported and extended the conclusions generated from the 24-hpf fate map. In the hyoid arch, crest cells that will contribute to the aHM and SY regions are tightly packed masses directly adjacent to the first pouch prior to chondrogenesis.

In order to understand the cellular basis for the losses of the aHM and SY regions in *integrinα5* mutants, we made time-lapse recordings of hyoid crest development in *integrinα5^−^; fli1*-GFP embryos from 38 hpf to 86 hpf (Video S3; Figure 7M–7R). Whereas in wild-type animals anterior hyoid crest cells were tightly packed masses adjacent to the first pouch at 38 hpf, in *integrinα5^−^; fli1*-GFP embryos, crest cells in the dorsal, anterior portion of the hyoid arch were more loosely packed (Figure 7M′). Strikingly, over the next day the crest-derived cells migrated out of the dorsal, anterior region of the mutant hyoid arch (enlarged in Figure 7N′–7Q′). By 86 hpf, the pHM cartilage region had formed, yet no *fli1*-GFP-positive cells were seen anterior to pHM (Figure 7R). Thus, we found a strong correlation between the lack of compaction and stabilization of dorsal, anterior hyoid crest cells and the loss of the aHM cartilage region in *integrinα5^−^; fli1*-GFP embryos.

## Discussion

### Isolation of a Zebrafish *integrinα5* Mutant

In this work, we isolated and characterized a zebrafish mutant allele *(b926)* that has variably penetrant and expressive reductions of the first pouch and hyoid aHM and SY cartilage regions. By positional mapping, allele segregation, and morpholino phenocopy, we identified the genetic basis of the lesion as a missense mutation in the ligand-binding domain of Integrinα5. As similar pharyngeal phenotypes were observed in mutant and morpholino-treated animals, we conclude that *b926* is a loss-of-function allele of *integrinα5*. However, we do not know if Integrinα5 activity is completely eliminated in *b926*. In addition, we observed strong maternal expression of *integrinα5* that could mitigate the zygotic loss of *integrinα5* in *b926.* Thus, the variable penetrance and expressivity of the *integrinα5* phenotype could be due to partial activity of mutant Integrinα5 or the presence of maternally supplied Integrinα5. Additionally, other integrins may act redundantly with Integrinα5 in pharyngeal development. A survey of nearly finished genome sequence (http://www.ensembl.org/Danio_rerio/) has uncovered at least 15 additional Integrin α chains, for which no expression or phenotypic data are known in zebrafish. Lastly, *integrinα5* is expressed strongly in many tissues, such as the otic placode, for which no overt phenotypes were observed in *b926*. Future studies, in particular those using animals in which both maternal and zygotic *integrinα5* have been eliminated, may reveal new functions of Integrinα5 in zebrafish development.

### The First Pouch Is Required for the Development of a Subset of Hyoid Cartilage

Our results point to an important role for the first pouch in the development of specific hyoid cartilage regions. We have used the incomplete penetrance of the pouch and cartilage phenotypes of *integrinα5^−^* animals to show that early first pouch defects are strongly predictive of later hyoid cartilage defects. Furthermore, transplantation experiments show that wild-type endoderm, but not crest, rescues first pouch and hyoid cartilage development in *integrinα5* mutants. We infer that Integrinα5 functions in the pharyngeal endoderm for the formation of the first pouch, and that the first pouch, in turn, interacts with postmigratory neural crest to promote cartilage development in a region of the hyoid arch. A role for the first endodermal pouch in promoting regional hyoid cartilage development is consistent with work in chicken showing that domains of pharyngeal endoderm specify region-specific cartilage shapes (Couly et al. 2002; Ruhin et al. 2003). Our data extend these findings, arguing that the formation of the first pouch is a critical step in allowing pharyngeal endoderm to interact with hyoid crest and promote the development of specific cartilage regions, aHM and SY. It will be interesting to see the extent to which the ability of different pharyngeal endoderm domains to induce cartilage elements of specific shapes depends on their ability to form discrete morphological structures such as the first pouch.

**Figure 8:**
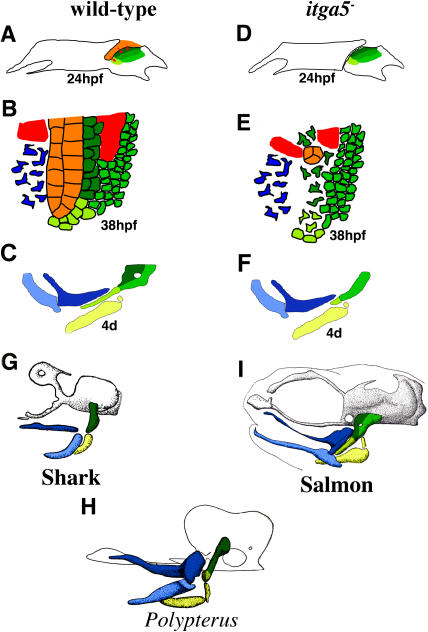
Model for Development and Evolution of Hyoid Cartilage (A–F) Models of hyoid development in wild-type (A–C) and *integrinα5^−^* (D–F) animals show the structure of hyoid arches at 24 hpf (A and D) and 38 hpf (B and E) and mandibular and hyoid cartilages at 4 d (C and F). (A) At 24 hpf of wild-type development, crest that will form aHM (dark green), pHM (medium green), and SY (light green) cartilage regions occupy distinct domains within the hyoid arch. Signals (red arrows) from the first pouch (orange) stabilize adjacent aHM- and SY-producing crest. (B) At 38 hpf of wild-type development, aHM- and SY-producing crest tightly pack along the first pouch. Cranial mesoderm (red) and some mandibular crest (blue) are also shown. (C) At 4 d of wild-type development, the HS cartilage is a composite of aHM, pHM, and SY regions. Also shown are the hyoid CH (yellow) and mandibular Meckel's (light blue) and palatoquadrate (dark blue) cartilages. (D) In *integrinα5^−^* animals, the first pouch is missing or very reduced at 24 hpf. (E) By 38 hpf, as a consequence of the lack of a first pouch, aHM and SY progenitors are disorganized and undergo gradual apoptosis. In contrast, the development of pHM progenitor cells does not require the first pouch. (F) At 4 d, aHM and SY cartilage regions are selectively reduced in *integrinα5^−^* animals. (G–I) The HS element has undergone extensive change during vertebrate evolution. In the illustrations (adapted from De Beer [1937]), the neurocranium is grey or outlined in black and mandibular and hyoid cartilages are color-coded as described above. Based on relations to morphological landmarks and data presented here on the tripartite mosaic development of HS, an evolutionary scheme is proposed. (G) In the dogfish shark *Scyliorhinus canicula,* a single rod-shaped element corresponds to pro-aHM/SY regions. (H) In the basal actinopterygian *Polypterus senegalus,* separate aHM and SY regions are present. (I) As shown for salmon, during actinopterygian evolution a new region, pHM, develops posterior to and fuses with aHM to create a wide HM plate that articulates with the neurocranium and supports an enlarged, overlying opercular apparatus (not shown).

How might the first pouch control development of specific cartilage regions within the hyoid arch? The first pouch forms at a time when hyoid crest cells are migrating to ventrolateral positions to form the hyoid arch (Veitch et al. 1999; J.G.C. and C.B.K., unpublished data). Upon reaching the developing arch, crest cells become less motile and form tightly packed masses adjacent to the first pouch. Our wild-type fate map shows that the hyoid cartilage regions that are lost in *integrinα5* mutants, aHM and SY, develop from crest cells immediately adjacent to the first pouch (Figure 8A). Our time-lapse recordings of wild-type cartilage development show that crest cells that will form aHM remain a tightly packed mass as the aHM region chondrifies (Figure 8B and 8C). In contrast, in *integrinα5* mutants, dorsal anterior hyoid crest cells are aberrantly motile and do not contribute to cartilage, whereas more posterior dorsal hyoid crest cells contribute normally to pHM (Figure 8D–8F). In addition, we observe increased apoptosis in the dorsal, anterior domain of *integrinα5^−^* hyoid arches from 25 to 35 hpf. Importantly, increased death of postmigratory hyoid crest cells was seen only when the first pouch was reduced. It will be interesting to examine whether the increased apoptosis observed in the dorsal anterior hyoid arches of *integrinα5^−/−^* mice (Goh et al. 1997) is a secondary consequence of a missing first pouch as well. In contrast to *integrinα5^−/−^* mice, in which an increase in cell death was seen earlier during crest migration, we found no evidence for increased death of migratory crest in *integrinα5^−^* zebrafish. However, our analysis cannot rule out that increased crest death during migratory stages may contribute to the infrequent, most severe cartilage phenotypes seen in *integrinα5^−^* embryos. Indeed, given the strong expression of *integrinα5* in migratory hyoid crest, future studies that further reduce Integrinα5 activity, for example by removing its maternal component, may uncover crest-autonomous functions of zebrafish Integrinα5 in the survival and/or migration of hyoid crest cells.

Our data show that the first pouch is required for the stabilization and survival of crest cells that will become aHM and SY. Interactions between the first pouch and adjacent hyoid crest could involve direct adhesion and/or diffusible signaling molecules. aHM and SY cartilage regions develop from crest-derived cells immediately adjacent to the first pouch at 24 hpf, whereas the pHM region develops from crest three cell diameters away. The remarkable specificity of the *integrinα5* cartilage phenotype suggests that pouch-derived signals act very locally, perhaps through cell–cell contract, to promote development of aHM and SY regions. In support of this, explant studies in the newt show that physical contact between pharyngeal endoderm and neural crest cells is necessary to promote the compaction and differentiation of crest into cartilage (Epperlein and Lehmann 1975). On the other hand, a signaling role for pharyngeal endoderm in crest survival also has been shown. In zebrafish, Fgf3 produced from the pharyngeal pouches acts as a secreted survival factor for neural crest (David et al. 2002; Nissen et al. 2003).

In addition to promoting stabilization and survival, might endoderm also control local gene expression in hyoid crest? In a small fraction of *integrinα5^−^* embryos, *gsc* expression domains in the hyoid arches were present but disorganized. However, it is possible that the aberrant *gsc* expression reflects a disorganization of the hyoid arch and not ectopic gene expression. Although disorganized *gsc* expression may correlate with more severe *integrinα5* phenotypes such as hyoid cartilage fusions (see Figure 2D), the significantly lower penetrance of *gsc* phenotypes compared to cartilage phenotypes implies that disorganized *gsc* expression is not the major cause of the specific cartilage defects. In addition, *hoxA2* expression was unaffected in the hyoid arches of *integrinα5^−^* embryos. Thus, although additional markers of hyoid crest need to be examined in *integrinα5^−^* embryos, we have found no strong evidence for the first pouch controlling gene expression in neighboring crest. Instead, our data argue that the first pouch locally controls cartilage development by promoting the compaction and survival of immediately adjacent crest-derived cells.

### Integrin-Mediated Outgrowth of the First Pharyngeal Pouch

We have shown that zebrafish *integrinα5* is expressed in pharyngeal endoderm during pouch formation and is required in the endoderm for development of the first pouch. The specificity for the first pouch of the *integrinα5* phenotype could be due to either redundancy with other integrins that function preferentially in posterior pouches or greater sensitivity of the first pouch to loss of integrin function. Although the most common phenotype in *integrinα5* mutants is loss of just the first pouch, we do occasionally see reductions of more posterior pouches as well, suggesting that Integrinα5 functions in the formation of most or all pouches. Moreover, as the more posterior pouches are required to segment the posterior crest mass into the five branchial arches from which the CB cartilages develop (Piotrowski and Nusslein-Volhard 2000), the variable loss of these pouches likely explains the reductions and rare fusions of CB cartilages seen in some *integrinα5^−^* animals.

How might Integrinα5 control pouch formation? The elaboration of a relatively uniform tissue into an organ of more complex curvature and ramifications, termed branching morphogenesis, is a common developmental program in both vertebrates and invertebrates. The formation of an iterative series of outpocketings, or pouches, from the pharyngeal endoderm can be thought of as analogous to branching morphogenesis. Integrins have well-documented roles in cell migration that could promote the outgrowth of branches (reviewed in Bokel and Brown 2002). From our unpublished observations in zebrafish, we know that pouches form by the directed lateral migration of pharyngeal endodermal cells (unpublished data). In this work, we find that *integrinα5* is expressed transiently in pouch-forming regions of pharyngeal endoderm and is required in endoderm for pouch formation. One possibility is that Integrinα5 adhesion promotes the lateral migration of endodermal cells to form pouches. Alternatively, Integrinα5 may be required for the specification or survival of pharyngeal endoderm that forms pouches. Future time-lapse imaging studies, in which pharyngeal endoderm morphogenesis is analyzed directly in *integrinα5^−^* embryos, will help to clarify the role of Integrinα5 in pouch formation.

### A Hierarchy of Tissue Interactions Control Regional Development in the Pharyngeal Arches

The exquisite functionality of the vertebrate jaw and pharynx requires the precise developmental coordination of their component parts. Arch-specific patterns of muscle connect with pharyngeal skeletal elements and are innervated by motor neurons of appropriate axial levels to orchestrate behaviors such as feeding and gill pumping. In *integrinα5* mutants, we see specific defects not only in the endodermal pouches and crest-derived cartilages, but also in cranial muscles and their associated motor nerves (schematized in Figure 2R). Both a dorsal mandibular (do) and a dorsal hyoid (ah) muscle are reduced in *integrinα5* mutants, and facial nerve VII, which innervates ah and other hyoid muscles, is reduced and/or fails to make a characteristic branch into two main fascicles. However, it is likely that muscle and nerve defects in *integrinα5* mutants are secondary to pouch and cartilage defects. Whereas *integrinα5* is expressed in endoderm and crest during pharyngeal morphogenesis, we were unable to detect *integrinα5* expression in cranial mesoderm or hindbrain neurons during axon outgrowth. In addition, muscle and nerve defects in *integrinα5* mutants were of significantly lower penetrance than the first pouch and hyoid cartilage defects. The low penetrance of the muscle and nerve defects might be explained by the variably expressive loss, in *integrinα5* mutants, of the pouch- and/or crest-derived signals on which muscle and nerve development depend. Unfortunately, due to the low penetrance of both muscle and nerve defects in *integrinα5* mutants, we were unable to directly test the tissue autonomy of these defects.

Our analysis of the *integrinα5* mutant does not distinguish between roles for endoderm and crest in patterning cranial muscle and nerves. The mesodermal cores that give rise to do and ah, the muscles affected in *integrinα5^−^* animals, lie close to and on opposite sides of the first pouch during pharyngeal arch development. The first endodermal pouch could have an early organizing role for cranial mesoderm. However, increasing evidence suggests that crest has a major role in patterning cranial mesoderm. Analysis of the *chinless* mutation in zebrafish has shown that *chinless* functions nonautonomously within the crest to promote muscle development (Schilling et al. 1996). In classic experiments in chicken, grafting of mandibular crest into more posterior arches can reprogram both skeletal and muscular fates (Noden 1983), though recent work suggests this effect is mediated by an isthmus-organizing activity included in the grafts (Trainor et al. 2002). In the larval zebrafish, do and ah are found in close association with the aHM region that is lost in *integrinα5* mutants. It is possible that loss of dorsal anterior hyoid crest in *integrinα5* mutants results in reductions not only of the aHM cartilage region but also of crest-derived signals that support development of do and ah muscles. As has been proposed by others (Noden 1983; Kontges and Lumsden 1996), the development of cranial muscles may depend less on their arch origin and more on the crest-derived structures, such as the aHM cartilage region and associated connective tissue, onto which they attach. Likewise, the reduction of facial nerve VII in *integrinα5* mutants could be due to either reductions in hyoid muscles and their associated survival signals or reductions in nerve outgrowth–promoting cues normally produced by the crest and/or endoderm. In conclusion, we see evidence for a local hierarchy of interactions that control the development of a specific region of the head encompassing dorsal hyoid and mandibular elements. At the top of the hierarchy is the endoderm-derived first pouch that promotes the development of a subset of hyoid crest into cartilage; in turn, this subset of hyoid crest may control development of neighboring muscles and, directly or indirectly, their associated nerve.

### Mosaic Assembly of Hyoid Cartilage: Implications for Evolution

The shape of the dorsal hyoid cartilage element has undergone extensive modification during actinopterygian evolution. In sharks (Figure 8G) and basal ray-finned fishes such as the bichir, Polypterus senegalus (Figure 8H), HM is a rod and SY is absent or not well elongated (De Beer 1937). In teleosts, highly derived ray-finned fish, the dorsal hyoid cartilage consists of a wider HM plate and a long SY extension (Figure 8I). The elaboration of HM and SY regions before teleosts emerged may have served to more efficiently support the jaw and increase gill pumping. The origin of the HM plate has long been a subject of debate. Allis (1915) proposed that the teleost HM plate consists of two regions that become fused together, whereas Edgeworth (1926), based on his staging series of the bowfin *Amia,* a relative of teleosts, concluded that the HM plate develops from a single anterior region that undergoes posterior growth to form a plate. There is ample precedence for differential growth as a mechanism of morphological change. For example, beautiful interspecies mosaic experiments have shown that the difference in beak length between ducks and quails is due to an autonomous growth potential of mandibular crest (Schneider and Helms 2003). However, our data support the composite two-region HM theory of Allis. We see a clear genetic dissociation between the development of aHM and pHM. Whereas aHM is absent in the majority of *integrinα5* mutants, pHM and the connecting opercle bone are still present even in the most severe class of *integrinα5* mutants. In addition, our fate mapping data show that, although aHM and pHM form a seamless HM plate in the larva, their progenitor cells occupy distinct, albeit contiguous, domains within the hyoid arch at 24 hpf, a result inconsistent with the posterior growth hypothesis of Edgeworth. Thus, aHM and pHM regions develop from spatially distinct domains of crest that depend on different sources of inductive signals.

Our data show that the first endodermal pouch is required for the development of aHM, yet in mutants that lack all pharyngeal endoderm, such as *casanova,* both aHM and pHM are lost (David et al. 2002). Thus, other structures of the pharyngeal endoderm besides the first pouch may be required for the development of pHM. One attractive possibility is that, whereas the first pouch induces aHM in the anterior part of the hyoid arch, the second pouch induces pHM in the posterior part of the arch. Allis (1915) concluded, based on relationships to morphological landmarks, that the rod-shaped HM cartilage in *Polypterus* represents the anterior portion of the teleost HM plate (i.e., aHM). Although more embryological studies of *Polypterus* need to be done, the evolution of the HM plate in ray-finned fishes such as teleosts appears to have involved a new induction event that led to a new region, pHM, being added to an older region, aHM. We propose that the de novo addition of regions to the skeletal pattern represents another mechanism, in addition to differential growth, of generating skeletal diversity during evolution.

## Materials and Methods

### 

#### Zebrafish strains and mutant screen.

Zebrafish *(Danio rerio)* raised at 28.5 °C were staged as previously described (Kimmel et al. 1995; Westerfield 1995). The wild-type line used was AB. *fli1*-GFP albino transgenic fish are the same as *TG(fli1:EGFP)^y1^; alb^b4^* (Lawson and Weinstein 2002), and *islet1*-GFP fish are as described (Higashijima et al. 2000). For the cartilage screen, ENU-mutagenized F2 parthenogenic diploid fish were generated by early pressure treatment (Streisinger et al. 1981; Solnica-Krezel et al. 1994) and fixed and stained with Alcian at 4 d. The *b926* allele was outcrossed to the AB strain and subsequently crossed onto the *fli1*-GFP and *islet1*-GFP backgrounds.

#### *integrinα5* identification and morpholino.

*b926* was mapped with respect to polymorphic microsatellites based on the hyoid cartilage phenotype. Initial mapping was performed on an AB background, and fine mapping was performed on an *islet1*-GFP background selected for a high degree of LG23 polymorphisms with respect to AB. Full-length *integrinα5* cDNA was obtained by 5′- and 3′-RACE. Standard molecular biological techniques were used. For genotyping, primers were designed to turn *b926* into a codominant polymorphism digestible with XmnI (GC156, TGACTGTGACCTTCAGCTCAATGTAAACGC; GC158, TGGATCTGGCCACCCACTGAGGTCGAAAAG). A morpholino (Genetools, Philomath, Oregon, United States) was designed against the exon13-intron splice site of *integrinα5* (*itga5*-MO) with the following sequence: ATGCTTTCTCACCTGGGTAGCCATT. Embryos were pressure-injected with 5 nl of 2 mg/ml *itga5*-MO as previously described (Maves et al. 2002).

#### Phenotypic analysis.

Alcian Green staining was performed as described (Miller et al. 2003). For flat mount dissections, Alcian-stained animals were digested for 1 h in 8% trypsin at 37 °C and transferred to 100% glycerol. Cartilages were dissected free from surrounding tissues with fine stainless-steel insect pins and photographed using a Zeiss (Oberkochen, Germany) Axiophot 2 microscope. Image background was cleaned up with Adobe Photoshop. For immunocytochemistry, embryos were prepared as described (Maves et al. 2002). Antibodies were used at the following dilutions: rabbit anti-GFP, 1:1,000 (Molecular Probes, Eugene, Oregon, United States); Zn-8, 1:400 (Trevarrow et al. 1990); MF-20, 1:10 (Developmental Studies Hybridoma Bank, University of Iowa, Iowa City, Iowa, United States); goat anti-rabbit Alexa Fluor 488 and anti-mouse Alexa Fluor 568 (Alexa568), both 1:300 (Molecular Probes). TUNEL staining was performed on embryos that were fixed overnight in 4% PFA, MeOH-permeabilized for 20 min, rehydrated, and treated with ProteinaseK (Sigma, St. Louis, Missouri, United States) for 2–20 min at room temperature. TdT/Fluorescein-dUTP reaction (Roche, Basel, Switzerland) was performed for 1 h on ice, followed by 1 h at room temperature. After labeling with Fluorescein-dUTP, immunocytochemistry was performed using rabbit anti-Fluorescein F_ab_ fragments (1:20,000, Molecular Probes) and goat anti-rabbit Alexa Fluor 568 antibodies (1:200, Molecular Probes). GFP fluorescence survived the procedure.

Probe syntheses and whole-mount in situ hybridizations were performed as previously described (Westerfield 1995). Embryos were mounted in glycerol and photographed using a Zeiss Axiophot 2 microscope. *integrinα5* RNA probes were made from plasmids pINT4150 and pINT4853, constructed by inserting RT-PCR fragments corresponding to nucleotides 254–2,207 and 1,679–2,960 of the *integrinα5* cDNA, respectively, into the TA vector (Invitrogen, Carlsbad, California, United States). Plasmids were linearized with BamHI, and T7 RNA polymerase was used for probe synthesis. Both probes gave identical expression patterns, and pINT4853 was used for photographs. *pea3* (Brown et al. 1998) and *gsc* (Schulte-Merker et al. 1994) probes were prepared as described, and mutant embryos were PCR genotyped.

#### Single-cell microelectroporation.

The microelectroporation technique was similar to that described by Lyons et al. (2003). *fli1*-GFP embryos, 24 hpf, were dechorionated, anesthetized with tricane solution, and bathed in a solution of 5 mg/ml pronase (Sigma) for 1 min to allow passage of the microelectrode through the skin. Agar mounting of embryos on slides was performed as described in Westerfield (1995). Under 50× Nomarski optics a micropipette filled with Alexa Fluor 568 dextran amines (Molecular Probes) was positioned next to the cell of interest, and a ground electrode was placed in the bath next to the embryo. Pulses of current between 1 and 4 uA were used to mobilize the dye.

Shortly after electroporation, the location of labeled cells relative to the *fli1*-GFP-expressing hyoid arch was assessed using confocal microscopy. Only embryos with one or two adjacently labeled cells were used in the analysis. Three-dimensional projections were constructed to determine the position of labeled cells in the arch relative to landmarks. All cell distances were made from the mid point of the cell to the landmark. Distance of the labeled cell from the edge of the first pouch was used to determine A-P position. Similarly, distances of labeled cells from the dorsal and lateral edges of the arch were used to determine D-V and mediolateral positions. In electroporations where two adjacent cells were labeled, the positions of each cell were measured and averaged. To control for variation in arch dimensions among individuals, measurements along the three axes were normalized to total axes lengths. At 4 d, embryos were imaged again to determine the fate of labeled cells in hyoid cartilage. The aHM region was defined as the anterior portion of HM that is characteristically lost in *integrinα5* mutants. pHM comprises the rest of the HM region. The SY region begins at the point of attachment to pHM. All graphing and statistical analysis were done using JMP (2002, SAS Institute, Cary, North Carolina, United States).

#### Time-lapse analysis and confocal imaging.

Embryos were manually dechorionated, anesthetized with tricane solution, transferred to 0.2% agarose in embryo media with 10 mM HEPES and tricane, and then mounted onto a drop of 3% methylcellulose on a rectangular coverslip with three superglued #1 square coverslips on each side. A ring of vacuum grease was added around the embryo to make an airtight seal upon addition of the top coverslip. A heated stage kept the embryos at 28.5 °C. Approximately 80-μm Z-stacks at 2-μm intervals were captured every 10 min using a Zeiss LSM 5 Pascal confocal fluorescence microscope. Movies of individual Z-sections were made by manually following cells and concatenating sections; further processing was done with Adobe Premiere. For single time point confocal sections, embryos were mounted without vacuum grease.

#### Endoderm and crest transplants.

Transplant techniques were as described (Maves et al. 2002). For endoderm transplants, donor embryos were injected at the one-cell stage with a mixture of 2% Alexa Fluor 568 dextran and 3% lysine-fixable biotin dextran (10,000 MW, Molecular Probes) along with TAR* RNA prepared according to David et al. (2002). At 40% epiboly (ca. 4 hpf), donor TAR* tissue was moved to the margins of *fli1*-GFP host embryos. For the crest transplants, donor *fli1*-GFP embryos were injected at the one-cell stage with the Alexa Fluor 568 mixture (“Alexa568”). At shield stage (ca. 6 hpf), donor tissue was taken from the animal cap and moved to a position approximately two germ ring widths from the margin and 90° from dorsal in a *fli1*-GFP host embryo. Confocal images of host embryos were captured at 38 hpf and 4 d, and embryos were subsequently fixed and Alcian-stained to visualize cartilages. For endoderm transplants, only embryos in which donor tissue contributed to the second and at least one other pouch were included in the analysis. For crest transplants, only embryos in which at least half the hyoid arch was composed of donor tissue were included in the analysis.

## Supporting Information

Video S1 Wild-Type Development of SY Cartilage Confocal time-lapse recording shows hyoid cartilage development in a wild-type *fli1*-GFP animal from 38 hpf to 86 hpf. At the beginning of the video, the mandibular (1) and hyoid (2) arches are numbered and an arrow denotes the normal position of the first pouch. At the end of the video (see Figure 7 for representative still images), the SY cartilage is pseudocolored blue. A red arrowhead denotes a cluster of cells adjacent to the first pouch that undergo cellular rearrangements and form the long anterior SY extension in wild-type animals.
(6.0 MB MOV).

Video S2 Wild-Type Development of HM Cartilage Confocal time-lapse recording shows hyoid cartilage development in a wild-type *fli1-*GFP animal from 38 hpf to 86 hpf. This video is a different depth of the same time-lapse recording as Video S1. At the beginning of the video, the mandibular (1) and hyoid (2) arches are numbered and an arrow denotes the normal position of the first pouch. At the end of the video (see Figure 7 for representative still images), the HM cartilage is pseudocolored blue, and the ao/lo muscle quadrant and opercle bone are pseudocolored red and purple, respectively, for reference. A red arrowhead points to hyoid crest–derived cells immediately adjacent to the first pouch that give rise to the aHM cartilage region in wild-type animals.
(7.1 mB MOV).

Video S3 Development of HM Cartilage in *integrinα5* Mutants Confocal time-lapse recording shows hyoid cartilage development in an *integrinα5^−^; fli1*-GFP animal from 38 hpf to 86 hpf. At the beginning of the video, the mandibular (1) and hyoid (2) arches are numbered and an arrow denotes the normal position of the first pouch. At the end of the video (see Figure 7 for representative still images), the HM cartilage is pseudocolored blue, and the ao/lo muscle quadrant and opercle bone are pseudocolored red and purple, respectively, for reference. A red arrowhead points to hyoid crest cells that display increased motility in *integrinα5^−^* animals and do not contribute to cartilage as in wild-type.
(6.0 KB MOV).

### Accession Numbers

The Genbank (http://www.ncbi.nlm.nih.gov) accession number for the zebrafish *integrinα5* cDNA is AY550244. Accession numbers for the related Integrinα5 proteins described in Figure 1 are Homo sapiens (Genbank P08648), Mus musculus (Genbank P11688), Xenopus laevis (Genbank Q06274), and Fugu rubripes (manually assembled from Fugublast M002304).

## Abbreviations

A-P: anterior-posterior
ah: adductor hyomandibulae
aHM: anterior hyomandibula
ao: adductor operculi
CB: ceratobranchial
CH: ceratohyal
D-V: dorsal-ventral
do: dilatator operculi
GFP: green fluorescent protein
*gsc*: *goosecoid*
HM: hyomandibula
hpf: hours post fertilization
HS: hyosymplectic
*itga5*: *integrinα5*
LG: Linkage Group
lo: levator operculi
pHM: posterior hyomandibula
RT-PCR: reverse transcription polymerase chain reaction
s: somites
SY: symplectic
TAR*: activated Taram-A receptor

## References

[pbio-0020244-Alexander1] Alexander J, Rothenberg M, Henry GL, Stainier DY (1999). casanova plays an early and essential role in endoderm formation in zebrafish. Dev Biol.

[pbio-0020244-Allis1] Allis EP (1915). The homologies of the hyomandibula of the gnathostome fishes. J Morphol.

[pbio-0020244-Bader1] Bader BL, Rayburn H, Crowley D, Hynes RO (1998). Extensive vasculogenesis, angiogenesis, and organogenesis precede lethality in mice lacking all alpha v integrins. Cell.

[pbio-0020244-Bokel1] Bokel C, Brown NH (2002). Integrins in development: Moving on, responding to, and sticking to the extracellular matrix. Dev Cell.

[pbio-0020244-Brown1] Brown LA, Amores A, Schilling TF, Jowett T, Baert JL (1998). Molecular characterization of the zebrafish PEA3 ETS-domain transcription factor. Oncogene.

[pbio-0020244-Couly1] Couly G, Creuzet S, Bennaceur S, Vincent C, Le Douarin NM (2002). Interactions between Hox-negative cephalic neural crest cells and the foregut endoderm in patterning the facial skeleton in the vertebrate head. Development.

[pbio-0020244-David1] David NB, Saint-Etienne L, Tsang M, Schilling TF, Rosa FM (2002). Requirement for endoderm and FGF3 in ventral head skeleton formation. Development.

[pbio-0020244-De1] De Beer GR (1937). The development of the vertebrate skull.

[pbio-0020244-Delannet1] Delannet M, Martin F, Bossy B, Cheresh DA, Reichardt LF (1994). Specific roles of the alpha V beta 1, alpha V beta 3 and alpha V beta 5 integrins in avian neural crest cell adhesion and migration on vitronectin. Development.

[pbio-0020244-Desban1] Desban N, Duband JL (1997). Avian neural crest cell migration on laminin: Interaction of the alpha1beta1 integrin with distinct laminin-1 domains mediates different adhesive responses. J Cell Sci.

[pbio-0020244-Edgeworth1] Edgeworth FH (1926). On the hyomandibula of Selachii, Teleostomi and *Ceratodus*. J Anat.

[pbio-0020244-Edgeworth2] Edgeworth FH (1935). The cranial muscles of vertebrates.

[pbio-0020244-Epperlein1] Epperlein HH, Lehmann R (1975). The ectomesenchymal-endodermal interaction-system (EEIS) of Triturus alpestris in tissue culture. I. Observations on the differentiation of visceral cartilage. Differentiation.

[pbio-0020244-Gardner1] Gardner H, Kreidberg J, Koteliansky V, Jaenisch R (1996). Deletion of integrin alpha 1 by homologous recombination permits normal murine development but gives rise to a specific deficit in cell adhesion. Dev Biol.

[pbio-0020244-Goh1] Goh KL, Yang JT, Hynes RO (1997). Mesodermal defects and cranial neural crest apoptosis in alpha5 integrin-null embryos. Development.

[pbio-0020244-Higashijima1] Higashijima S, Hotta Y, Okamoto H (2000). Visualization of cranial motor neurons in live transgenic zebrafish expressing green fluorescent protein under the control of the islet-1 promoter/enhancer. J Neurosci.

[pbio-0020244-Hughes1] Hughes GM, Shelton G (1958). The mechanism of gill ventilation in three freshwater teleosts. J Exp Biol.

[pbio-0020244-Hunt1] Hunt P, Gulisano M, Cook M, Sham MH, Faiella A (1991). A distinct Hox code for the branchial region of the vertebrate head. Nature.

[pbio-0020244-Kil1] Kil SH, Krull CE, Cann G, Clegg D, Bronner-Fraser M (1998). The alpha4 subunit of integrin is important for neural crest cell migration. Dev Biol.

[pbio-0020244-Kimmel1] Kimmel CB, Ballard WW, Kimmel SR, Ullmann B, Schilling TF (1995). Stages of embryonic development of the zebrafish. Dev Dyn.

[pbio-0020244-Kimmel2] Kimmel CB, Miller CT, Keynes RJ (2001). Neural crest patterning and the evolution of the jaw. J Anat.

[pbio-0020244-Kontges1] Kontges G, Lumsden A (1996). Rhombencephalic neural crest segmentation is preserved throughout craniofacial ontogeny. Development.

[pbio-0020244-Lawson1] Lawson ND, Weinstein BM (2002). In vivo imaging of embryonic vascular development using transgenic zebrafish. Dev Biol.

[pbio-0020244-Le1] Le Douarin NM (1982). The neural crest.

[pbio-0020244-Lyons1] Lyons DA, Guy AT, Clarke JD (2003). Monitoring neural progenitor fate through multiple rounds of division in an intact vertebrate brain. Development.

[pbio-0020244-Maves1] Maves L, Jackman W, Kimmel CB (2002). FGF3 and FGF8 mediate a rhombomere 4 signaling activity in the zebrafish hindbrain. Development.

[pbio-0020244-Miller1] Miller CT, Yelon D, Stainier DY, Kimmel CB (2003). Two endothelin 1 effectors, hand2 and bapx1, pattern ventral pharyngeal cartilage and the jaw joint. Development.

[pbio-0020244-Mould1] Mould AP, Askari JA, Humphries MJ (2000). Molecular basis of ligand recognition by integrin alpha 5beta 1. I. Specificity of ligand binding is determined by amino acid sequences in the second and third NH2-terminal repeats of the alpha subunit. J Biol Chem.

[pbio-0020244-Nissen1] Nissen RM, Yan J, Amsterdam A, Hopkins N, Burgess SM (2003). Zebrafish foxi one modulates cellular responses to Fgf signaling required for the integrity of ear and jaw patterning. Development.

[pbio-0020244-Noden1] Noden DM (1983). The role of the neural crest in patterning of avian cranial skeletal, connective, and muscle tissues. Dev Biol.

[pbio-0020244-Piotrowski1] Piotrowski T, Nusslein-Volhard C (2000). The endoderm plays an important role in patterning the segmented pharyngeal region in zebrafish *(Danio rerio)*. Dev Biol.

[pbio-0020244-Piotrowski2] Piotrowski T, Ahn DG, Schilling TF, Nair S, Ruvinsky I (2003). The zebrafish van gogh mutation disrupts tbx1, which is involved in the DiGeorge deletion syndrome in humans. Development.

[pbio-0020244-Platt1] Platt JB (1893). Ectodermic origin of the cartilages of the head. Anat Anz.

[pbio-0020244-Roehl1] Roehl H, Nusslein-Volhard C (2001). Zebrafish pea3 and erm are general targets of FGF8 signaling. Curr Biol.

[pbio-0020244-Ruhin1] Ruhin B, Creuzet S, Vincent C, Benouaiche L, Le Douarin NM (2003). Patterning of the hyoid cartilage depends upon signals arising from the ventral foregut endoderm. Dev Dyn.

[pbio-0020244-Schilling1] Schilling TF, Kimmel CB (1994). Segment and cell type lineage restrictions during pharyngeal arch development in the zebrafish embryo. Development.

[pbio-0020244-Schilling2] Schilling TF, Kimmel CB (1997). Musculoskeletal patterning in the pharyngeal segments of the zebrafish embryo. Development.

[pbio-0020244-Schilling3] Schilling TF, Walker C, Kimmel CB (1996). The chinless mutation and neural crest cell interactions in zebrafish jaw development. Development.

[pbio-0020244-Schneider1] Schneider RA, Helms JA (2003). The cellular and molecular origins of beak morphology. Science.

[pbio-0020244-Schulte-Merker1] Schulte-Merker S, Hammerschmidt M, Beuchle D, Cho KW, De Robertis EM (1994). Expression of zebrafish goosecoid and no tail gene products in wild-type and mutant no tail embryos. Development.

[pbio-0020244-Solnica-Krezel1] Solnica-Krezel L, Schier AF, Driever W (1994). Efficient recovery of ENU-induced mutations from the zebrafish germline. Genetics.

[pbio-0020244-Springer1] Springer TA (1997). Folding of the N-terminal, ligand-binding region of integrin alpha-subunits into a beta-propeller domain. Proc Natl Acad Sci U S A.

[pbio-0020244-Streisinger1] Streisinger G, Walker C, Dower N, Knauber D, Singer F (1981). Production of clones of homozygous diploid zebra fish *(Brachydanio rerio)*. Nature.

[pbio-0020244-Testaz1] Testaz S, Duband JL (2001). Central role of the alpha4beta1 integrin in the coordination of avian truncal neural crest cell adhesion, migration, and survival. Dev Dyn.

[pbio-0020244-Trainor1] Trainor PA, Ariza-McNaughton L, Krumlauf R (2002). Role of the isthmus and FGFs in resolving the paradox of neural crest plasticity and prepatterning. Science.

[pbio-0020244-Trevarrow1] Trevarrow B, Marks DL, Kimmel CB (1990). Organization of hindbrain segments in the zebrafish embryo. Neuron.

[pbio-0020244-Veitch1] Veitch E, Begbie J, Schilling TF, Smith MM, Graham A (1999). Pharyngeal arch patterning in the absence of neural crest. Curr Biol.

[pbio-0020244-Warga1] Warga RM, Nusslein-Volhard C (1999). Origin and development of the zebrafish endoderm. Development.

[pbio-0020244-Westerfield1] Westerfield M (1995). The zebrafish book.

[pbio-0020244-Weston1] Weston JA, Yoshida H, Robinson V, Nishikawa S, Fraser ST (2004). Neural crest and the origin of ectomesenchyme: Neural fold heterogeneity suggests an alternative hypothesis. Dev Dyn.

[pbio-0020244-Whittaker1] Whittaker CA, DeSimone DW (1993). Integrin alpha subunit mRNAs are differentially expressed in early *Xenopus* embryos. Development.

[pbio-0020244-Yang1] Yang JT, Rayburn H, Hynes RO (1993). Embryonic mesodermal defects in alpha 5 integrin-deficient mice. Development.

[pbio-0020244-Yang2] Yang JT, Rayburn H, Hynes RO (1995). Cell adhesion events mediated by alpha 4 integrins are essential in placental and cardiac development. Development.

[pbio-0020244-Yang3] Yang JT, Bader BL, Kreidberg JA, Ullman-Cullere M, Trevithick JE (1999). Overlapping and independent functions of fibronectin receptor integrins in early mesodermal development. Dev Biol.

